# Born Under COVID-19 Pandemic Conditions: Infant Regulatory Problems and Maternal Mental Health at 7 Months Postpartum

**DOI:** 10.3389/fpsyg.2021.805543

**Published:** 2022-01-26

**Authors:** Anna Perez, Ariane Göbel, Lydia Yao Stuhrmann, Steven Schepanski, Dominique Singer, Carola Bindt, Susanne Mudra

**Affiliations:** ^1^Division of Neonatology and Pediatric Intensive Care, Center for Obstetrics and Pediatrics, University Medical Center Hamburg-Eppendorf, Hamburg, Germany; ^2^Department of Child and Adolescent Psychiatry, Psychotherapy and Psychosomatics, University Medical Center Hamburg-Eppendorf, Hamburg, Germany; ^3^Division of Experimental Feto-Maternal Medicine, Department of Obstetrics and Fetal Medicine, University Medical Center Hamburg-Eppendorf, Hamburg, Germany; ^4^Institute of Developmental Neurophysiology, Center for Molecular Neurobiology Hamburg (ZMNH), University Medical Center Hamburg-Eppendorf, Hamburg, Germany

**Keywords:** COVID-19, SARS-CoV-2, maternal mental health, infant regulatory problems, parenthood

## Abstract

**Background:**

The SARS-COVID-19 pandemic and its associated disease control restrictions have in multiple ways affected families with young children, who may be especially vulnerable to mental health problems. Studies report an increase in perinatal parental distress as well as symptoms of anxiety or depression in children during the pandemic. Currently, little is known about the impact of the pandemic on infants and their development. Infant regulatory problems (RPs) have been identified as early indicators of child socio-emotional development, strongly associated with maternal mental health and the early parent–infant interaction. Our study investigates whether early parenthood under COVID-19 is associated with more maternal depressive symptoms and with a perception of their infants as having more RPs regarding crying/fussing, sleeping, or eating, compared to mothers assessed before the pandemic.

**Methods:**

As part of a longitudinal study, 65 women who had given birth during the first nationwide disease control restrictions in Northern Germany, were surveyed at 7 months postpartum and compared to 97 women assessed before the pandemic. RPs and on maternal depressive symptoms were assessed by maternal report. Number of previous children, infant negative emotionality, and perceived social support were assessed as control variables.

**Results:**

Compared to the control cohort, infants born during the COVID-19 pandemic and those of mothers with higher depressive symptoms were perceived as having more sleeping and crying, but not more eating problems. Regression-based analyses showed no additional moderating effect of parenthood under COVID-19 on the association of depressive symptoms with RPs. Infant negative emotionality was positively, and number of previous children was negatively associated with RPs.

**Limitations:**

Due to the small sample size and cross-sectional assessment, the possibility for more complex multivariate analysis was limited. The use of parent-report questionnaires to assess infant RPs can support but not replace clinical diagnosis.

**Conclusions:**

The pandemic conditions affecting everyday life may have a long-term influence on impaired infant self- and maternal co-regulation and on maternal mental health. This should be addressed in peripartum and pediatric care. Qualitative and longitudinal studies focusing on long-term parental and infant outcomes under ongoing pandemic conditions are encouraged.

## Introduction

The ongoing SARS-CoV-2 pandemic has had profound global effects on economy, healthcare systems, and societal structures (World Bank, 2020; Whitehead et al., 2021). The experience of nationwide lockdowns, including school closures, quarantine measures, prolonged social isolation, and reduced possibility for recreational and outdoor activities, puts children and adolescents at a higher risk for mental health problems (Fegert et al., 2020). Increased distress and irritability, anxiety and depressive symptoms, reduced quality of life, and disrupted sleeping routines have been reported in children and adolescents living under pandemic-related restrictions (Crescentini et al., 2020; Jiao et al., 2020; Loades et al., 2020; Nearchou et al., 2020; MacKenzie et al., 2021; Ravens-Sieberer et al., 2021b). A growing public health concern is the potential impact of the SARS-CoV-2 pandemic on vulnerable populations like pregnant women, postpartum mothers, and young infants (Almeida et al., 2020; Caparros-Gonzalez and Alderdice, 2020; Thapa et al., 2020; McDonald et al., 2021). Disruption of healthcare services and fear of attending health-care facilities may additionally affect the wellbeing of mothers and their babies (Burki, 2020; Khalil et al., 2021). Many studies have described poorer maternal mental health outcomes during versus before the pandemic, such as elevated depressive and anxiety symptoms, symptoms of posttraumatic stress disorder, and sleep disorders (Berthelot et al., 2020; Hessami et al., 2020; Wu et al., 2020; Zanardo et al., 2020; Xie et al., 2021).

The changed conditions many families face under COVID-19 restrictions may negatively affect their everyday life and exacerbate the dynamics between maternal psychological distress and infant emotional and behavioral problems, particularly in families with preexisting risk factors (Burki, 2020; Prime et al., 2020; Asbury et al., 2021; Ravens-Sieberer et al., 2021a; Thorell et al., 2021). Thus, investigating these associations in early infancy is crucial for understanding the potential long-lasting effects of the pandemic on maternal and infant mental health.

### Infant Regulatory Problems and Maternal Mental Health

Regulatory problems (RPs) such as excessive infant crying, sleeping or eating problems show the strongest prevalence rates in young infants up to 18 months (Skovgaard et al., 2007; Lyons-Ruth et al., 2017; Georg et al., 2021). Infant self-regulation as one of the fundamental developmental tasks during the first years is, besides maturation processes, strongly associated with co-regulation capacities of the primary caregivers, embedded in a mutual, dyadic interaction (Berger et al., 2007; Tronick and Beeghly, 2011; Beeghly et al., 2016). Thus, clinical features of RPs comprise a complex interplay between parental, infant and interactional factors such as infant behavioral problems in at least one developmental area, caregiver overload syndrome, and dysfunctional interaction patterns in dealing with infant problems (“symptom trias”; Cierpka, 2015; Georg et al., 2021).

According to the clinical classification system DC: 0–5, infants are diagnosed with primary “Sleep, Eating and Crying Disorders,” if the functioning of the infant, family, or both, is persistently impaired, and other diagnoses such as a sensory processing disorder are ruled out (ZERO TO THREE, 2016). Although often of transient nature (Cierpka, 2015), RPs may persist in certain infants with long-term consequences such as cognitive, behavioral, and emotional difficulties across childhood, including emotional and attention problems, anxiety or depression (Canivet et al., 2000; Hemmi et al., 2011; Schmid and Wolke, 2014; Winsper and Wolke, 2014; Smarius et al., 2017; Cook et al., 2020). Risk factors for infant RPs comprise perinatal influences (Alvik et al., 2011; Bilgin and Wolke, 2016), infant factors such as “difficult temperament” and contextual factors (Martini et al., 2017). According to the reciprocal character of the early parent–infant-relationship, not only can RPs lead to parental distress, but parental psychopathology or maladaptive emotion regulation may in turn affect infants’ regulatory capacity and are further risk factors for the emergence of infant RPs (Canivet et al., 2005; Petzoldt et al., 2016).

In the first years of life, infants are especially vulnerable to an unfavorable environment and dysfunctional family dynamics due to their strong dependency on their caregivers and to rapid brain and behavioral development (Lyons-Ruth et al., 2017). Regarding maternal mental health, perinatal depression and anxiety disorders have been identified as risk factors for problems in infant socio-emotional development and, more specifically, in regulatory behavior (Goodman and Gotlib, 1999; Van den Bergh and Marcoen, 2004; Feldman et al., 2009; Granat et al., 2017; Bates et al., 2020). Prevalence rates for postnatal maternal depression (10–20%) and anxiety (15%) highlight the relevance of maternal mental health problems during early motherhood (Gavin et al., 2005; Gelaye et al., 2016; Dennis et al., 2017).

In the literature, perinatal maternal depression was – individually and in comorbidity with anxiety – associated with excessive infant crying and sleeping problems (Petzoldt et al., 2016; Martini et al., 2017; Toffol et al., 2019). More eating problems have also been reported in relation to elevated maternal anxiety and depression scores (Coulthard and Harris, 2003; Martini et al., 2017). Maternal depressive symptoms in the prenatal period may further interact with the level of postnatal symptomatology in their effect on RPs in infancy and early childhood (Toffol et al., 2019; Schultz et al., 2020; Dias and Figueiredo, 2021). There is further evidence for divergent associations of RPs with anxious and depressive symptoms depending on the onset of maternal symptomatology. A systematic review found infant excessive crying associated with concurrent and subsequent but not preceding maternal depressive symptoms, and further with preceding and concurrent but not subsequent maternal anxiety (Petzoldt, 2018). Additionally, general prenatal anxiety and fear of childbirth were reported to predict infant difficult temperament (Thiel et al., 2020). Continuing difficulties in the mother–child interaction may further increase the risk for parental distress and depression as well as for persistent infant RPs (Papoušek et al., 2004; Hemmi et al., 2011; Lyons-Ruth et al., 2017).

### Regulatory Problems and Maternal Mental Health Under Pandemic Conditions

The pandemic as an early familial stressful experience with the potential to affect birth, postpartum socio-emotional wellbeing, and the parental ability to co-regulate the baby, may be also associated with unfavorable regulatory outcomes in early infancy. Exposure to stressors can lead to cognitive, emotional and physical fatigue in parents which in turn may strain the parent–child relationship (Deater-Deckard, 2004).

Several studies indicated an increased risk for postpartum depression in mothers who delivered their babies during the pandemic (Wu et al., 2020; Hui et al., 2021; Madera et al., 2021). In a German study, higher birth-related anxieties and lower emotional bonding to the fetus were then reported for women pregnant during the COVID-19 outbreak (Schaal et al., 2021). Others reported higher parental stress levels in new parents during than before the pandemic (Brown et al., 2020; Taubman-Ben-Ari et al., 2021). RPs during the pandemic may be promoted and encountered by a less favorable maternal interaction and co-regulation behavior, due to elevated levels of parental stress and depression and multiple pandemic-induced concerns of everyday life, potentially increasing problematic child behavior and the likelihood of family conflict escalation (Prime et al., 2020).

In Hamburg, Germany, COVID-19 related disease control regulations led in March 2020 to the closing of schools and kindergartens, cultural, sport, and recreational facilities, substantial contact restrictions in the public and private domain, along with official recommendations for remote working from home, whenever possible. These restrictions were partially eased during summer 2020 and reinforced with a nationwide lockdown in November 2020. Schools and kindergartens were then reopened in August 2020, mostly part-time enforcing strong hygiene and social distance regulations. Hence, the second wave of the pandemic beginning in autumn 2020 (City of Hamburg, 2021) with rising incidences was associated with public concerns, while many families were still struggling with the consequences of the first lockdown. Thus far, no previous studies have investigated the wellbeing of young parents and their infants in Northern Germany during ongoing restrictions compared to families pre-COVID-19.

Our study aimed to assess both aspects, infant RPs regarding crying/fussing, eating, and sleeping, and maternal depressive symptoms in the context of parenthood under COVID-19 conditions, compared to a sample of mother–child dyads assessed before the worldwide outbreak of SARS-CoV-2. We expected that the pandemic-related difficulties parents might experience within the first 7 months postpartum may lead to more maternal depressive symptoms and more infant RPs in families during COVID-19 and that the association of maternal depressive symptoms with infant RPs would be stronger in the COVID-19 than in the control cohort.

Moreover, infant negative emotionality, maternal perceived social support, and number of previous children were also considered due to their potential influence on RPs. Specifically, more difficult infant temperament and lower social support reported by the mother have been previously linked with more infant RPs (Papoušek et al., 2009; Martini et al., 2017). Number of previous children also seemed relevant in the specific context of the pandemic as mothers with more than one child in the family were affected by restrictions in multiple domains of everyday life with social distancing, the closing of schools, kindergartens, and recreational facilities impacting older children’s daily routines and leading to crowding inside the home space (Fegert et al., 2020; Prime et al., 2020).

In a previous analysis, slightly higher levels of generalized anxiety were found in the COVID-19 cohort at 3 weeks postpartum, compared to the control cohort (Perez et al., 2021). According to a previously reported association between maternal anxiety and infant RPs (Martini et al., 2017; Petzoldt, 2018) and considering the high rate of comorbidity between anxiety and depression (Falah-Hassani et al., 2017), we explored whether generalized anxiety had an individual effect beyond depressive symptoms on infant outcome. Finally, as the current literature indicates, breastfeeding behavior may change under pandemic conditions (Brown and Shenker, 2021; Latorre et al., 2021; Sakalidis et al., 2021). Therefore, a potential effect of current breastfeeding status on infant RPs was explored.

## Materials and Methods

### Study Sample and Procedure

As part of a longitudinal study on parental mental health and infant socio-emotional development and adjustment under the ongoing SARS-CoV-2 pandemic, maternal reports on sociodemographic and obstetric background, psychological and infant variables were assessed at 3 weeks (T1) and again at 6 to 7 (T2) and 12 months (T3) postpartum. All participants of the COVID-19 cohort had given birth at the University Medical Center Hamburg-Eppendorf between March 15th and May 1st, 2020, i.e., within the first 6 weeks of the nationwide disease control restrictions in the federal state of Hamburg. The control cohort comprised a population-based prospective longitudinal pregnancy study sample at the same institution (PAULINE – “Prenatal Anxiety and Infant Early Emotional Development”) who had participated under pre-pandemic conditions (Göbel et al., 2020; Mudra et al., 2020). For both cohorts, inclusion criteria were age 18 or older, sufficient German language skills, and singleton birth. Respondents with chronic infections, substance abuse or severe pregnancy complications, and mothers of babies born premature (<37th week of pregnancy) or with low birth weight (<2,500 g) were excluded. For the current study, T2 data were included and collected between early 2016 and early 2019 for the control cohort and between October and November 2020 for the COVID-19 cohort. Written informed consent was obtained from all participants. Ethical approval was obtained from the ethical committee of the Hamburg Medical Chamber (PV5574).

Participants of the control cohort were recruited upon initial presentation for their birth registration at the University Medical Center in the third trimester of pregnancy. For the COVID-19 cohort, eligible women were recruited 3–8 weeks after their hospital discharge via personal phone call based on contact information from admission charts. For both cohorts, the same set of self-report questionnaires were sent out by post. Of the 90 women in the COVID-19 cohort who participated at 3 weeks postpartum, 65 responded at 6–7 months postpartum (72.2%). In the control cohort, data of 97 (101 participants at T1 corresponding to 96.0%) participants was available at 6–7 months postpartum. The higher dropout rate in the COVID-19 cohort could be related to the varied study setting with no direct personal contact with the study team due to the prevailing circumstances of the pandemic. Additionally, a habituation effect toward the pandemic-related restrictions over time may have also reduced interest in further study participation.

### Variables and Instruments

#### Infant Regulatory Problems

Regulatory problems based on parent report with reference to a typical week in the family’s life were assessed with the Questionnaire for Crying, Feeding, and Sleeping (German: Fragebogen zum Schreien, Füttern, Schlafen, SFS; Groß et al., 2013). The first three items of the questionnaire refer to Wessel’s “rule of threes” (ZERO TO THREE, 2016), used as indicative for a clinically relevant level of excessive crying and included in the current study for descriptive purposes only: in a typical week, does the infant (a) cry for 3 h or more, (b) on three or more days per week and (c) for three consecutive weeks? Further, it comprises a scale of 24 items covering RPs in the context of sleeping, fussing, and crying (hereinafter labeled as “sleeping/crying”), a scale of 13 items in the context of eating/feeding, and another scale with a focus on co-regulatory strategies of the parents. For the current study, the scales “sleeping/crying” and “feeding” were analyzed as outcome variables. Items are answered on a 4-point scale, ranging from 1 to 4. Higher scores indicate higher levels of RPs. In the current analysis, scale reliability was good for the “sleeping/crying” scale (Cronbach’s α = 0.86) and satisfying for the “feeding” scale (α = 0.79).

#### Maternal Depressive Symptoms

Maternal level of depressive symptoms during the past 7 days was assessed with the Edinburgh Postnatal Depression Scale (EPDS; Cox et al., 1987; Bergant et al., 1998). Ten items are scored on a 4-point scale, ranging from 0 to 3, resulting in a total maximum score of 30. A higher score indicates a higher level of depressive symptoms. Current research identified a cut-off value of 11 with maximized sensitivity and specificity indicating an elevated risk for a mild depressive disorder in pregnant and postpartum women. A cut-off of 13 was identified to show most specificity (Levis et al., 2020). Scale reliability in the current study was good (α = 0.83).

#### Infant Negative Emotionality

Infant negative emotionality was measured with the Infant Behavior Questionnaire (IBQ; Rothbart, 1986; Pauli-Pott et al., 2003), comprising five dimensions of infant temperament via parent report. In the current study, the scales “distress to novelty” (10 items) and “distress to limitations” (17 items) were combined to cover a broader dimension of infant negative emotionality (Rothbart, 1986; Worobey and Blajda, 1989; Mertesacker et al., 2004). Items are rated on a 7-point scale. The mean scale score of this composite score (27 items) can range between 1 to 7, with a higher score indicating more negative emotionality. In the current study, reliability for this composite score was good (α = 0.80).

#### Maternal Perceived Social Support

Perceived social support on an emotional and instrumental level was assessed with a subscale of the Berlin Social Support Scale (BSSS; Schulz and Schwarzer, 2003). The scale consists of 8 items, which are rated on a 4-point scale. Mean scale scores are calculated, ranging from 1 to 4, with a higher score indicating higher levels of perceived social support. In the current study, scale reliability was excellent (α = 0.90).

#### Generalized Anxiety

As part of an explorative analysis, generalized anxiety was assessed with the Generalized Anxiety Disorder Scale (GAD-7; Spitzer et al., 2006). The scale consists of 7 items, which are rated on a 4-point scale. Mean scale scores range from 0 to 3. Scale reliability in the current study was excellent (α = 0.85).

### Statistical Analysis

First, descriptive statistics of the continuous variables (mean, standard deviation, and range) and bivariate correlations were investigated. Group differences for relevant variables were reported based on univariate ANOVA, Mann–Whitney-*U*-Test, and χ*^2^*-Test, where applicable. The research question was investigated with two regression-based moderation analyses, with infant RPs (1) in the context of sleeping and crying and (2) in the context of feeding as outcome variables. In both analyses, maternal depressive symptoms were included as predictor variable, and giving birth under pandemic conditions (0 = control cohort vs. 1 = COVID-19 cohort) as moderator variable. Infant negative emotionality, maternal perceived social support, and number of previous children were included as control variables. Also, regression analyses were repeated without the interaction term to explore the main effects. In subsequent explorative analyses, effects of maternal anxiety and breastfeeding status were further investigated. Missing values in the included variables (max 7%) were replaced using Expectation Maximization estimation. Moderation analyses were conducted with generalized linear models with prior bootstrapping for robust testing. Effects were considered significant at *p* < 0.05, two-tailed. Effect sizes were estimated based on eta squared for bivariate group differences (η^2^) and partial eta squared ($\eta_{\text{p}}^{2}$) for regression analysis, with values of 0.01 indicating a small-sized, 0.06 a medium-sized, and 0.14 a large-sized effect. Regarding statistical power for the regression analyses, at least *n* = 98 cases in the models with the interaction term and *n* = 92 cases in the models without the interaction term were needed to identify an expected minimum overall effect of medium size with a power of 80% and a significance level of *p* < 0.05 (G*Power 3.1.9.6). For all statistical analyses, IBM SPSS Statistics for Windows (Version 27) was used.

## Results

### Descriptive Statistics and Preliminary Analyses

Characteristics of the final sample included in this analysis are listed in Table 1. Overall, participants in both cohorts were well educated and had an average to high household income. Education level (*U* = 2705.00, *Z* = −1.156, *p* = 0.248) and household income (*U* = 2604.00, *Z* = −1.279, *p* = 0.201) did not significantly differ between cohorts. The number of previous children born by the mother ranged between one to three and did not significantly differ between groups (*F* = 1.89, *p* = 0.172). Across cohorts, there were slightly more boys than girls. However, the distribution of child gender did not significantly differ between cohorts, χ^2^(1) = 1.08, *p* = 0.299. In both cohorts, the majority of mothers (80.0 and 64.9%, respectively) were still breastfeeding their infant at 6–7 months postpartum, with no statistical difference between cohorts, χ^2^(1) = 0.50, *p* = 0.481. Regarding age, mothers in the COVID-19 cohort were significantly older (Welch = 5.10, *p* < 0.05).

**TABLE 1:** Characteristics of the sample.

	PAULINE	PAULINE-COVID-19
	(*n* = 97)	(*n* = 65)
Maternal age at T1 (years), *M* (*SD*)	34.51 (3.25)	36.02 (4.55)
**Educational level, *n* (%)**		
No degree	1 (1)	0 (0.0)
Main or middle school	4 (4.1)	6 (9.3)
High school graduation	18 (18.6)	5 (7.7)
University degree	68 (70.1)	51 (78.5)
Information not provided	6 (6.2)	3 (4.6)
**Household income^a^, *n* (%)**		
≤1,000 €	2 (2.0)	1 (1.5)
1,001–2000 €	8 (8.3)	3 (4.6)
2,001–4,000 €	24 (24.8)	14 (21.6)
≥4,001 €	59 (60.9)	46 (70.8)
Information not provided	5 (5.2)	1 (1.5)
Number of previous children, *M, SD*	1.49 (0.58)	1.64 (0.74)
**Child sex assigned at birth, *n* (%)**		
Girl	35 (36.1)	29 (44.6)
Boy	61 (62.9)	35 (53.8)
Information not provided	1 (1.5)	1 (1.5)
**Breastfeeding status at T1, *n* (%)**		
Exclusive breastfeeding	15 (15.5)	12 (18.5)
Partial breastfeeding	48 (49.5)	40 (61.5)
Not breastfeeding	24 (24.7)	13 (20.0)
Information not provided	10 (10.3)	–

*T1 = 7 months postpartum.*

*^a^Household net income including child benefit.*

Table 2 lists the descriptive statistics of the included psychometric variables. Mean values in sleeping/crying were slightly and significantly higher in the COVID-19 cohort. From a clinical perspective, five infants (7.7%) in the COVID-19 and three infants (3.1%) in the control cohort fulfilled Wessel’s “rule of threes” criteria. Regarding eating/feeding, there was no significant difference between groups. Regarding the EPDS, 16.9% in the COVID-19 cohort and 8.2% in the control cohort reached the cut-off value of 11 or higher. Also, 12.3% in the COVID-19, and 5.1% in the control cohort reached a cut-off of 13 or higher indicating a mild depressive disorder (Levis et al., 2020). While group differences were statistically significant for the depressive symptom level, there were no significant group differences regarding infant negative emotionality, and perceived social support.

**TABLE 2:** Descriptive statistics of the included psychometric variables for the main analyses.

	COVID-19 (*n* = 65)				PAULINE (*n* = 97)				*F*	*p*	η^2^
	*M*	*SD*	Min	Max	*M*	*SD*	Min	Max			
RPs with sleeping/crying (SFS)	1.80	0.41	1.09	2.75	1.61	0.33	1.04	2.50	9.77	0.002	0.06
RPs with eating/feeding (SFS)	1.24	0.27	1.00	2.46	1.21	0.32	1.00	2.46	0.29	0.594	0.00
Maternal depressive symptoms (EPDS)	6.25	4.48	0	20.00	5.05	4.37	0	25.00	4.03	0.046	0.03
Infant negative emotionality (IBQ)	3.12	0.58	2.04	4.72	2.95	0.59	1.89	4.53	0.38	0.538	0.00
Maternal perceived social support (BSSS)	3.86	0.28	2.80	4.00	3.80	0.35	2.40	4.00	1.04	0.308	0.01

*SFS, German Questionnaire for Crying, Feeding and Sleeping; EPDS, Edinburgh Postnatal Depression Scale; IBQ, Infant Behavior Questionnaire; BSSS, Berlin Social Support Scale.*

Significant bivariate zero-order correlations (Table 3) of RPs in the context of both sleeping/crying and eating with the included variables were small to medium-sized, positively associated with maternal depressive symptoms and infant negative emotionality, and negatively associated with maternal perceived social support. A significant positive correlation with belonging to the COVID-19 cohort was found for RPs with sleeping/crying but not eating. Number of previous children was not significantly correlated with the included variables.

**TABLE 3:** Bivariate correlations of the included variables.

		1	2	3	4	5	6	7
(1)	RPs with sleeping/crying (SFS)	–	0.29**	0.25**	0.33**	–0.16	0.52**	−0.25**
(2)	RPs with eating/feeding (SFS)		–	–0.04	0.18*	–0.02	0.44**	−0.22**
(3)	Cohort^a^			–	0.16*	0.11	0.05	–0.08
(4)	Maternal depressive symptoms (EPDS)				–	–0.06	0.14	−0.34**
(5)	Number of previous children					–	–0.02	0.02
(6)	Infant negative emotionality (IBQ)						–	–0.12
(7)	Maternal perceived social support (BSSS)							–

*RPs, regulatory problems; SFS, German Questionnaire for Crying, Feeding and Sleeping; EPDS, Edinburgh Postnatal Depression Scale; IBQ, Infant Behavior Questionnaire; BSSS, Berlin Social Support Scale.*

*^a^Cohort: 0 = control group, 1 = COVID-19 group.*

**p < 0.01, *p < 0.05, two-tailed.

### Effects of Cohort and Depressive Symptoms on Regulatory Problems

First, the interactive effect of cohort affiliation and depressive symptoms on infant RPs in the context of crying/sleeping was investigated, after controlling for number of previous children, infant negative emotionality and perceived social support. Moderation analysis did not show a significant relevance of being in the COVID-19 or control cohort for the association between depressive symptoms and infant RPs in the context of crying/sleeping *F*(1,155) = 0.58, *p* = 0.383, 95% CI [−0.01, 0.02]. Analysis was repeated without the interaction term to further investigate the main effects of the included variables. Results for the main effects are listed in Table 4. A significant, medium-sized effect of affiliation to the COVID-19 cohort on infant RPs in the context of sleeping/crying was found, *F*(1,156) = 10.68, *p* < 0.01. Also, maternal depressive symptoms had a positive, small to medium-sized effect on sleeping/crying RPs, *F*(1,156) = 7.82, *p* < 0.01. Thus, infants in the COVID-19 cohort and infants of mothers with higher depressive symptom levels showed more RPs regarding crying/sleeping. Concerning the control variables, women with more previous children reported their infant to show fewer RPs, *F*(1,156) = 5.98, *p* < 0.05. Moreover, infants with higher levels of negative emotionality showed more RPs in the context of crying/sleeping, *F*(1,156) = 54.97, *p* < 0.01. Maternal perceived social support did not significantly explain variance in RPs in the context of crying/sleeping, *F*(1,156) = 2.89, *p* = 0.078.

**TABLE 4:** Main effects for the associations of infant RPs with the included variables (*n* = 162).

	RPs with sleeping/crying					RPs with eating/feeding				
	*B*	*SE B*	*p*	95% CI	$\eta_{\text{p}}^{2}$	*B*	*SE B*	*p*	95% CI	$\eta_{\text{p}}^{2}$
Constant	1.76	0.06	0.001	[1.65, 1.88]	0.85	1.22	0.06	0.001	[1.11, 1.33]	0.73
Cohort^a^	0.15	0.05	0.003	[0.06, 0.25]	0.06	0.05	0.04	0.186	[−0.03, 0.13]	0.01
Maternal depressive symptoms (EPDS)	0.02	0.01	0.006	[0.01, 0.03]	0.05	0.01	0.01	0.204	[−0.00, 0.02]	0.01
Number previous children	–0.09	0.04	0.013	[−0.16, −0.02]	0.04	0.01	0.03	0.796	[−0.05, 0.07]	0.00
Infant negative emotionality (IBQ)	0.01	0.00	0.001	[0.01, 0.02]	0.26	0.01	0.00	0.001	[0.00, 0.01]	0.18
Maternal perceived social support (BSSS)	–0.13	0.07	0.078	[−0.29, 0.01]	0.02	–0.15	0.09	0.104	[−0.34, 0.04]	0.03

*CI, confidence interval for B, based on bias-corrected and accelerated (BCa) bootstrap interval; EPDS, Edinburgh Postnatal Depression Scale; IBQ, Infant Behavior Questionnaire; BSSS, Berlin Social Support Scale.*

*^a^Cohort: 0 = control group, 1 = COVID-19 group.*

Second, the interaction effect of the cohort affiliation and maternal depressive symptoms on infant RPs in the context of eating problems was investigated. Again, there was no significant cohort effect on the association between depressive symptoms and infant RPs regarding infant eating/feeding, *F*(1,155) = 0.414, *p* = 0.489, 95% CI [−0.01, 0.03]. Subsequent analysis excluding the interaction term showed no significant main effect of cohort, *F*(1,156) = 1.54, *p* = 0.185 or depressive symtoms on the outcome, *F*(1,156) = 1.45, *p* = 0.215 (for details, see Table 4). Of the control variables, only infant negative emotionality had a significant positive, large-sized effect on infant eating/feeding problems, *F*(1,156) = 34.72, *p* < 0.001. The effects for number of previous children, *F*(1,156) = 0.09, *p* = 0.796, and perceived social support *F*(1,156) = 4.51, *p* = 0.104 were not significant. Thus, only infants with more negative emotionality were reported to have more RPs in the context of eating/feeding.

### Explorative Analyses

In a previous analysis, the COVID-19 cohort reported slightly but significantly higher levels of generalized anxiety compared to the control group at 3 weeks postpartum (Perez et al., 2021), and anxiety symptoms have shown independent associations with infant RPs in previous studies (Martini et al., 2017; Petzoldt, 2018). Therefore, we further explored whether generalized anxiety was additionally associated with RPs, with the results showing non-significant effects on crying/sleeping, *F*(1,155) = 0.33, *p* = 0.531, 95% CI [−0.02, 0.02], and on infant eating, *F*(1,155) = 2.51, *p* = 0.212, 95% CI [−0.01, 0.04].

Finally, a potential effect of current breastfeeding status on infant RPs was explored. An interaction between belonging to the COVID-19 cohort and breastfeeding (0 = not breastfeeding, 1 = exclusively or partly breastfeeding) was not found for RPs regarding infant crying/sleeping, *F*(1,142) = 0.23, *p* = 0.976, 95% CI [−0.25, 0.25], or eating, *F*(1,142) = 1.04, *p* = 0.315, 95% CI [−0.32, 0.09]. Subsequent analysis focusing on the main effects showed a significant small, positive effect of breastfeeding status on RPs in the context of crying/sleeping, *F*(1,144) = 2.84, *p* < 0.05, 95% CI [−0.22, −0.02], $\eta_{\text{p}}^{2}$ = 0.03. Thus, mothers not breastfeeding at T2 reported their infant to have less crying/sleeping problems. There was no significant effect of breastfeeding on RPs regarding infant eating, *F*(1,144) = 0.11, *p* = 0.751, 95% CI [−0.13, 0.10].

### Sensitivity Analysis

Results in the main and explorative analyses were confirmed when (a) only participants without missing data were included (*n* = 143) and (b) additionally controlled for maternal age, maternal education, infant gender, or monthly household income (for more detailed information, see Supplementary Table 1–3). Only the effect of breastfeeding status on infant crying/sleeping turned into a non-significant trend after including the control variables.

## Discussion

This study aimed to assess infant RPs regarding crying/fussing, eating, and sleeping as well as maternal depressive symptoms in the context of parenthood under COVID-19 conditions, compared to a sample of mother–child dyads assessed before the worldwide outbreak of SARS-CoV-2. Additionally, a potential effect of generalized anxiety and of breastfeeding status on infant outcome was explored.

### Regulatory Problems and the Pandemic

Mothers in the COVID-19 group and those with higher depressive symptoms reported more infant RPs regarding crying and sleeping than mothers in the control cohort. However, the association of depressive symptoms with infant RPs was not stronger in the COVID-19 group compared to the control group. This finding suggests that the observed independent effect of pandemic conditions on infant regulatory behavior may be explained by other aspects not analyzed in the current study. A potential mediator for this association is maternal pandemic-induced stress, as the imposed restriction measures affected multiple domains of families’ everyday life. In line with this, a recently published longitudinal study from Italy showed that the association of infants’ regulatory capacity at 3 months with maternal postnatal anxiety was mediated by parenting stress (Provenzi et al., 2021). With increased stress levels and potentially reduced abilities to mentalize and to recognize infants’ needs, parents may also become less capable of providing positive leadership within their family (McMahon and Meins, 2012). This again may increase the child’s negativity and thus the likelihood of escalation of family conflict or parental avoidance (Prime et al., 2020). Another recent study demonstrated that parents of infants with RPs showed more distress and more difficulty taking on their infant’s subjective perspective (Georg et al., 2018). Due to their close relationship during infancy, reciprocity between caregiver and infant may promote a vicious circle of negative interaction patterns, infant RPs, and caregivers’ mental health. Further, prenatal stress and depression associated with the pandemic may have already been present during pregnancy affecting infant ability to self-regulate, since there is wide evidence for long-term effects of prenatal stress, anxiety and depression on child development (Kingston et al., 2012; Korja et al., 2017; Buffa et al., 2018). Moreover, when considering COVID-19 as a potent prenatal stressor during pregnancy, a recent study (Manning et al., 2021) found a significant correlation between maternal stress perception and alterations in 3-month-old infant’s functional brain connectivity. Of note, neither stress nor respective proxies were assessed in the current investigation. Since the current study did not include infant observations or other sources than the maternal perception of infant behavior, we cannot rule out that the mothers’ perspective might be affected by COVID-related parenting distress with a potential effect on the parent–infant-interaction. Therefore, future studies should integrate longitudinal designs to distinguish transient from long-term consequences for mother and child.

### Regulatory Problems and Maternal Mental Health

Compared to the control cohort, more mothers in the COVID-19 cohort reported depressive symptom levels above the cut-off values indicative of mild depressive disorders. This observation is in line with the findings of several studies, who reported more depressive symptoms (Ostacoli et al., 2020; Achterberg et al., 2021) and a higher risk for postpartum depression (Davenport et al., 2020; Hessami et al., 2020; Madera et al., 2021) in mothers of young infants during the COVID-19 pandemic. During COVID-19, anxiety about one’s own and the family’s, including the new infant’s, health may be exacerbated by financial worries either directly through individual job loss or indirectly through uncertainty about the situation of the national economy and/or local unemployment (Prime et al., 2020). While in lockdown, these stressors may be further aggravated by social constraints, including isolation, decline in social support, and changes in work and daily family routine. Here, an important example would be working from home while school closings are enforced, leading to the division of the home space (Prime et al., 2020). Elevated depressive symptoms under COVID-19 conditions may also be a trajectory of elevated stress and anxiety levels during pregnancy, as most of the included mothers in the COVID-19 cohort experienced – at least the last part of – their pregnancy under pandemic conditions. The association of stressors present during pregnancy with an increased risk of developing anxious-depressive symptoms in new mothers has been described before (Leigh and Milgrom, 2008). Apart from that, transient depressive symptoms could also be a reaction to the exceptional pandemic conditions and the resulting restrictions affecting everyday life, with a potentially stronger influence on families (Prime et al., 2020), in particular those with preexisting risk for increased distress (Burki, 2020; Asbury et al., 2021; Ravens-Sieberer et al., 2021a; Thorell et al., 2021). In summary, our findings, together with those of others, highlight the need to monitor and support mothers and their infants during the pandemic from pregnancy, to childbirth and during the first postpartum months (Hessami et al., 2020) to assess potential consequences for families with young children longitudinally.

Maternal symptoms of depression were associated with more infant crying and/or sleeping problems in the current study. Maternal or parental depressive and anxiety disorders have been identified as predictors of infant RPs before, with evidence for specific effects of parental mental health symptoms on infant RPs. In a prospective longitudinal study excessive infant crying was specifically associated with maternal anxiety disorders, while infant sleeping problems were related to maternal depressive symptoms (Petzoldt et al., 2016; Martini et al., 2017). In our study, neither the pandemic nor maternal mental health seemed to influence infant eating regulation, which stands in contrast to other studies that described an effect of depression and/or anxiety on infant eating behavior (Coulthard and Harris, 2003; Petzoldt et al., 2016; Martini et al., 2017). Nonetheless, the relationship between maternal mental health and infant RPs may be moderated by other maternal factors. A previous study, also using the SFS, showed that infants of anxious mothers had more RPs regarding crying/sleeping and eating than infants of non-anxious mothers. The association between maternal anxiety and infant RPs regarding crying/sleeping was, however, moderated by positive maternal engagement in stressful situations (Richter and Reck, 2013).

### Regulatory Problems and Other Infant and Maternal Characteristics

Certain infant characteristics attributed to infant negative emotionality are often found in association with RPs, such as increased excitability, high activity level, lack of adaptability, and impaired self-soothing ability (Papoušek et al., 2009). Martini et al. (2017) described fussy/difficult infant temperament as an important factor associated with extensive infant crying, eating, and sleeping problems. In several other studies, infant negative temperament was identified as a predictor of sleep disturbance, with impeded self-soothing, an inability to adapt, diminished smiling, and reduced activity used as markers of infant negative temperament (Hirtz et al., 1993; Touchette et al., 2005; Heitkamp and Pauli-Pott, 2008; Weinraub et al., 2012; Sorondo and Reeb-Sutherland, 2015). Also, perceived infant negative emotionality in our study was associated with more sleeping and crying problems as well as more eating problems. The latter is in line with a positive association between infant temperament and eating difficulties in infancy reported previously (Wolke et al., 2009).

In both cohorts, women who had given birth to their first child reported more crying and sleeping problems. This may be due to a higher level of uncertainty and lack of experience in taking care of the new infant (Martinez-Galiano et al., 2019; Hines et al., 2021; Stuhrmann et al., 2021). However, others have reported no influence of number of previous pregnancies on RPs (Martini et al., 2017).

The current findings support the association between perceived crying/fussing and sleeping RPs and maternal depressive symptoms as reported by the participants themselves via questionnaire. In contrast to previous research (Petzoldt et al., 2016; Martini et al., 2017) no individual effect of anxiety symptoms on RPs was found in the subsequent explorative analysis. Notably, anxiety symptoms were assessed with the GAD-7, which is a well-established screening instrument for generalized anxiety symptoms but is not comparable to a diagnosis based on clinical interviews as used by the group of Petzoldt et al. (2016).

Perceived social support had no influence on infant RPs. Martini et al. (2017) have described a potential association of higher social support with lower probability of excessive infant crying, with all three domains of social support (emotional and practical, social integration) being relevant. However, previous findings on the effect of social support during the perinatal period have been mixed. An Irish study comparing stress levels, social support, health behaviors, and stress-reduction strategies for pregnant women before and during the SARS-CoV-2 pandemic reported that although perceived social support was significantly reduced during the pandemic, the level of pregnancy-related stress was not increased (Matvienko-Sikar et al., 2021). On the other hand, in a study from Australia, some women reported even more quality time with their family and increased partner support due to remote working restrictions (Sakalidis et al., 2021). These results indicate a complex interplay of the SARS-CoV-2 pandemic and individual consequences, also depending on preexisting conditions within the family.

Most (80%) of the mothers in the COVID-19 group were still breastfeeding their infants at 6–7 months of age, significantly more than in the control group. This is in line with a cross-sectional study reporting a higher rate of exclusive breastfeeding (82%) than typically observed among Australian women (Sakalidis et al., 2021). In the study by Sakalidis et al. (2021), continuation of exclusive breastfeed during the pandemic was explained by citing positive outcomes related to the lockdown, including less pressure, improved bonding with their infant, and more time with the family. Among breastfeeding mothers, crying, and sleeping problems of their infants were more pronounced compared to infants already weaned from the breast. In our study breastfeeding showed no effect on infant eating regulation. However, mothers who had stopped breastfeeding reported less RPs in the context of sleeping/crying. This is in line with previous studies, in which a positive association of breastfeeding with sleeping problems has been reported (Wolke et al., 1998; Sondergaard et al., 2000; Schmid et al., 2011; Galbally et al., 2013; Hysing et al., 2014). Overall, breastfeeding had opposite effects on different RPs. The more breastfeeding, the more sleeping problems the children had, and the less breastfeeding, the more eating problems have been reported (Schmid et al., 2011).

### Strengths and Limitations

The main strength of this investigation is the direct comparison of mothers of young infants during COVID-19 and under pre-pandemic conditions, implementing the same psychometric instruments at the same institution. However, there are several limitations to consider. Due to the modest sample size and the cross-sectional assessment, the possibility for more complex multivariate analysis was limited. The use of parent-report questionnaires to assess maternal symptoms of depression and infant RPs can support but not replace clinical interviews and diagnoses. A selection bias cannot be ruled out, as it was the participants’ own responsibility to return the questionnaires, especially in the COVID-19 cohort, which was less closely connected with the study team. It can further not be ruled out that an attrition bias may influence variance in the reported variables. Moreover, the relatively homogenous SES and the primary exclusion of infant complications may result in overseeing families with more socio-economic or medical constraints during the pandemic (Simha et al., 2020). Pandemic-related distress within the family increases when parents have children with special needs and/or challenging behaviors to care for. Pre-existing family characteristics (e.g., financial situation before COVID-19) also affect how vulnerable a family is to crises, as consequences of the pandemic may be greater for lower SES families (Prime et al., 2020; Ravens-Sieberer et al., 2021a).

## Conclusion

Overall, our results suggest that the pandemic conditions affecting multiple domains of families’ everyday life may have consequences for mothers’ mental health, maternal perception of impaired infant self-regulation and maternal co-regulation, and should be addressed in current postpartum maternal and pediatric care. Qualitative and longitudinal studies focusing on transient and long-term parental and infant outcomes under ongoing pandemic conditions are encouraged.

## Data Availability Statement

The datasets presented in this article are not readily available because of the ethical committee’s decision. Requests to access the datasets should be directed to a.goebel@uke.de or s.mudra@uke.de.

## Ethics Statement

The studies involving human participants were reviewed and approved by the Ethik-Kommission der Ärztekammer Hamburg. The patients/participants provided their written informed consent to participate in this study.

## Author Contributions

AP: study conceptualization, participant recruitment, data acquisition, investigation, and writing original draft. AG: conceptualization, methodology, formal analysis, and writing original draft. LS and SS: investigation, data curation, and writing original draft. DS: revision original draft and resources. CB: revision original draft, resources, and funding. SM: conceptualization, investigation, methodology, writing original draft, supervision, project administration, and funding. All authors contributed to the article and approved the submitted version.

## Conflict of Interest

The authors declare that the research was conducted in the absence of any commercial or financial relationships that could be construed as a potential conflict of interest.

## Publisher’s Note

All claims expressed in this article are solely those of the authors and do not necessarily represent those of their affiliated organizations, or those of the publisher, the editors and the reviewers. Any product that may be evaluated in this article, or claim that may be made by its manufacturer, is not guaranteed or endorsed by the publisher.
