# Supplementary material for: Born Under COVID-19 Pandemic Conditions: Infant Regulatory Problems and Maternal Mental Health at 7 Months Postpartum
Source: Front Psychol. 2022 Jan 26;12:805543. doi: 10.3389/fpsyg.2021.805543 (PMC8826543; doi:10.3389/fpsyg.2021.805543)
Supplement: Supplementary file 1 [file Data_Sheet_1.docx]

| **Supplementary Table 1. Sensitivity analyses for the main effects on associations of infant RPs, only cases with full data. ( (*n* = 143)** | | | | | | | | | | | | | | | |
| --- | --- | --- | --- | --- | --- | --- | --- | --- | --- | --- | --- | --- | --- | --- | --- |
|  | RPs w sleeping/crying | | | | | |  | RPs w eating/feeding | | | | | |  | |
|  | *B* | SE *B* | *p* | 95% CI | | η²_p_ |  | *B* | SE *B* | *p* | 95% CI | | η²_p_ |  |  |
| Constant | 1.91 | 0.80 | .000 | [1.73 | 2.01] | 0.83 |  | 1.27 | 0.06 | .001 | [1.10 | 1.33] | 0.72 |  |  |
| Cohort^a^ | 0.15 | 0.05 | .003 | [-0.26 | -0.05] | 0.06 |  | 0.05 | 0.04 | .230 | [-0.33, | 0.13] | 0.01 |  |  |
| Maternal depressive symptoms (EPDS) | 0.02 | 0.01 | .013 | [0.00 | 0.03] | 0.05 |  | 0.01 | 0.01 | .132 | [-0.00, | 0.02] | 0.01 |  |  |
| Number previous children | -0.09 | 0.04 | .019 | [-0.16 | -0.02] | 0.04 |  | 0.01 | 0.04 | .813 | [-0.06, | 0.09] | 0.00 |  |  |
| Infant negative emotionality (IBQ) | 0.01 | 0.00 | .001 | [0.01 | 0.01] | 0.25 |  | 0.01 | 0.00 | .001 | [0.00, | 0.01] | 0.18 |  |  |
| Maternal perceived social support (BSSS) | -0.15 | 0.08 | .073 | [-0.30 | 0.02] | 0.02 |  | -0.14 | 0.10 | .139 | [-0.34, | 0.04] | 0.03 |  |  |
| *Notes*. CI= confidence interval for *B*, based on bias-corrected and accelerated (BCa) bootstrap interval; EPDS, Edinburgh Postnatal Depression Scale; IBQ, Infant Behavior Questionnaire; BSSS, Berlin Social Support Scale.  ^a^ Cohort: 0 = control group, 1 = COVID-19 group. | | | | | | | | | | | | | | | |

| **Supplementary Table 2.**  **Sensitivity analyses for the main effects on associations of infant RPs with the included main variables and generalized anxiety (*n* = 162)** | | | | | | | | | | | | | | | |
| --- | --- | --- | --- | --- | --- | --- | --- | --- | --- | --- | --- | --- | --- | --- | --- |
|  | RPs w sleeping/crying | | | | | |  | RPs w eating/feeding | | | | | |  | |
|  | *B* | SE *B* | *p* | 95% CI | | η²_p_ |  | *B* | SE *B* | *p* | 95% CI | | η²_p_ |  |  |
| Constant | 1.65 | 0.28 | .001 | [1.09 | 2.20] | 0.18 |  | 0.90 | 0.22 | .001 | [0.45 | 1.35] | 0.09 |  |  |
| Cohort^a^ | 0.14 | 0.06 | .007 | [-0.25 | -0.04] | 0.06 |  | 0.06 | 0.04 | .194 | [-0.27, | 0.14] | 0.01 |  |  |
| Maternal depressive symptoms (EPDS) | 0.04 | 0.01 | .005 | [0.00 | 0.03] | 0.03 |  | 0.01 | 0.01 | .889 | [-0.01, | 0.02] | 0.02 |  |  |
| Number previous children | -0.08 | 0.04 | .026 | [-0.17 | -0.02] | 0.03 |  | 0.01 | 0.04 | .923 | [-0.07, | 0.07] | 0.00 |  |  |
| Infant negative emotionality (IBQ) | 0.01 | 0.00 | .001 | [0.01 | 0.01] | 0.25 |  | 0.01 | 0.00 | .001 | [0.00, | 0.01] | 0.19 |  |  |
| Maternal perceived social support (BSSS) | -0.15 | 0.09 | .091 | [-0.33 | 0.01] | 0.02 |  | -0.14 | 0.11 | .191 | [-0.35, | 0.06] | 0.03 |  |  |
| Generalized anxiety (GAD-7) | -0.01 | 0.01 | .053 | [-0.26 | 0.02] | 0.00 |  | -0.01 | 0.01 | .226 | [-0.01 | 0.39] | 0.01 |  |  |
| Household income | 0.01 | 0.02 | .656 | [-0.02 | 0.04] | 0.00 |  | 0.02 | 0.01 | .095 | [-0.01 | 0.04] | 0.02 |  |  |
| Maternal education level | 0.01 | 0.02 | .842 | [-0.04 | 0.05] | 0.00 |  | 0.01 | 0.03 | .626 | [-0.03 | 0.07] | 0.00 |  |  |
| Maternal age | 0.00 | 0.01 | .543 | [-0.01 | 0.02] | 0.00 |  | -0.00 | 0.01 | .772 | [-0.01 | 0.01] | 0.01 |  |  |
| Infant gender | 0.04 | 0.05 | .419 | [-0.07 | 0.15] | 0.01 |  | 0.04 | 0.05 | .371 | [-0.05 | 0.13] | 0.00 |  |  |
| *Notes*. CI= confidence interval for *B*, based on bias-corrected and accelerated (BCa) bootstrap interval; EPDS, Edinburgh Postnatal Depression Scale; IBQ, Infant Behavior Questionnaire; BSSS, Berlin Social Support Scale.  ^a^ Cohort: 0 = control group, 1 = COVID-19 group. | | | | | | | | | | | | | | | |

| **Supplementary Table 3.**  **Sensitivity analyses for the main effects on associations of infant RPs with the included main variables and breastfeeding status (*n* = 162)** | | | | | | | | | | | | | | | |
| --- | --- | --- | --- | --- | --- | --- | --- | --- | --- | --- | --- | --- | --- | --- | --- |
|  | RPs w sleeping/crying | | | | | |  | RPs w eating/feeding | | | | | |  | |
|  | *B* | SE *B* | *p* | 95% CI | | η²_p_ |  | *B* | SE *B* | *p* | 95% CI | | η²_p_ |  |  |
| Constant | 1.65 | 0.27 | .001 | [1.08 | 2.18] | 0.18 |  | 0.96 | 0.21 | .001 | [0.50 | 1.41] | 0.09 |  |  |
| Cohort^a^ | 0.14 | 0.06 | .014 | [-0.25 | -0.03] | 0.05 |  | 0.06 | 0.04 | .172 | [-0.31, | 0.14] | 0.01 |  |  |
| Maternal depressive symptoms (EPDS) | 0.04 | 0.01 | .005 | [0.00 | 0.03] | 0.04 |  | 0.01 | 0.01 | .142 | [-0.00, | 0.02] | 0.02 |  |  |
| Number previous children | -0.08 | 0.04 | .037 | [-0.15 | -0.02] | 0.03 |  | 0.01 | 0.03 | .701 | [-0.05, | 0.08] | 0.00 |  |  |
| Infant negative emotionality (IBQ) | 0.01 | 0.00 | .001 | [0.01 | 0.01] | 0.25 |  | 0.01 | 0.00 | .001 | [0.00, | 0.01] | 0.19 |  |  |
| Maternal perceived social support (BSSS) | -0.15 | 0.09 | .099 | [-0.33 | 0.02] | 0.04 |  | -0.16 | 0.10 | .093 | [-0.35, | 0.02] | 0.03 |  |  |
| Breastfeeding status | -0.10 | 0.05 | .054 | [-0.18 | 0.01] | 0.02 |  | -0.02 | 0.05 | .802 | [-0.02 | 0.15] | 0.01 |  |  |
| Household income | 0.01 | 0.02 | .541 | [-0.02 | 0.04] | 0.00 |  | 0.02 | 0.01 | .088 | [-0.01 | 0.04] | 0.02 |  |  |
| Maternal education level | 0.01 | 0.02 | .676 | [-0.04 | 0.05] | 0.00 |  | 0.01 | 0.03 | .626 | [-0.03 | 0.07] | 0.00 |  |  |
| Maternal age | 0.00 | 0.01 | .713 | [-0.01 | 0.02] | 0.00 |  | -0.00 | 0.01 | .746 | [-0.01 | 0.01] | 0.01 |  |  |
| Infant gender | 0.04 | 0.05 | .399 | [-0.06 | 0.14] | 0.01 |  | 0.05 | 0.05 | .315 | [-0.05 | 0.14] | 0.00 |  |  |
| *Notes*. CI= confidence interval for *B*, based on bias-corrected and accelerated (BCa) bootstrap interval; EPDS, Edinburgh Postnatal Depression Scale; IBQ, Infant Behavior Questionnaire; BSSS, Berlin Social Support Scale.  ^a^ Cohort: 0 = control group, 1 = COVID-19 group. | | | | | | | | | | | | | | | |
